# Perspectives on the ethical concerns and justifications of the 2006 Centers for Disease Control and Prevention HIV testing: HIV screening policy changes

**DOI:** 10.1186/1472-6939-14-46

**Published:** 2013-11-12

**Authors:** Michael J Waxman, Roland C Merchant, M Teresa Celada, Melissa A Clark

**Affiliations:** 1 Department of Emergency Medicine, Albany Medical College, Albany, USA; 2 Department of Emergency Medicine, Alpert Medical School, Providence, USA; 3 Department of Community Health, Alpert Medical School and Public Health Program of Brown University, Providence, USA; 4 Wheaton College, Wheaton, USA

## Abstract

**Background:**

The 2006 Centers for Disease Control and Prevention (CDC) revised recommendations for HIV testing in clinical settings contained seven specific changes to how health care facilities should provide HIV testing. These seven elements have been both supported and challenged in the lay and medical literature. Our first paper in BMC Medical Ethics presented an analysis of the three HIV testing procedural changes included in the recommendations. In this paper, we address the four remaining elements that concern HIV screening policy changes: (1) nontargeted HIV screening, (2) making HIV screening similar to screening for other treatable conditions, (3) increasing HIV screening without assured additional funding for linkage to care, and (4) making patients bear the costs of increased HIV screening in health care settings.

**Methods:**

We interviewed 25 members from the fields of US HIV advocacy, care, policy, and research about the ethical merits and demerits of the four changes to HIV screening policies. We performed a qualitative analysis of the participant responses in the interviews and summarized the major themes.

**Results:**

Participants commented that nontargeted HIV screening and making HIV screening similar to screening for other treatable medical conditions was ethical when it broadened the scope of people being tested for HIV. However, they believed it was unethical when it did not respect the exceptional nature of HIV and HIV testing. Some participants favored more testing regardless if there was assured additional funding for linkage to care or if patients might bear the costs of testing because they believed that merely alerting patients of their status was beneficial and would lead to positive consequences. Other participants found ethical flaws with testing without assured linkage to care and patients bearing the costs of testing, as this could discriminate against those who could not pay.

**Conclusions:**

Our findings suggest that there are fundamental ethical disagreements that shape views on CDC’s recommended HIV testing policies. Differences remain on whether or not HIV remains an exceptional condition that requires it to be treated differently than other treatable conditions. Disagreement also exists on the responsibilities of health care providers and rights of patients in regards to screening in (1) the absence of assured linkage to care after an HIV diagnosis and (2) paying for the costs of HIV screening. Resolution of these disagreements is needed to serve the common goal of using testing to facilitate medical care for those who are HIV infected and for reducing HIV transmission.

## Background

In September 2006, the Centers for Disease Control and Prevention (CDC) proffered their revised recommendations for HIV testing in health care settings [1]. Since the release of these recommendations, there has been much discussion in the lay, medical, and public health literature as to whether these recommendations are morally problematic [2-31]. Through a review of both the CDC’s recommendations and the commentaries on this topic in the lay and medical literature, we identified seven major elements or changes, either explicit in the recommendations themselves or implied by them, which were viewed by some as potentially morally troublesome. These changes were: (1) an opt-out approach to screening, (2) no separate signed consent for HIV testing, (3) optional prevention counseling at the time of testing, (4) nontargeted screening, (5) making HIV screening similar to screening for other treatable conditions, (6) increasing HIV screening without assured additional funding for linkage to care, and (7) making patients bear the costs of increased HIV screening in health care settings.

The first three of these recommendations – an opt-out approach, no separate signed consent for HIV testing, and optional prevention counseling at the time of testing – called for changes in how health care providers approach, consent, and counsel patients when testing for HIV. The ethical issues surrounding these topics were discussed in our previous article in BMJ Medical Ethics entitled, *Perspectives on the ethical concerns and justifications of the 2006 Centers for Disease Control and Prevention HIV testing recommendations*[32]. In this manuscript we address the four remaining changes that concern revised HIV testing policies: nontargeted HIV screening, making HIV screening similar to screening for other treatable medical conditions, increasing HIV screening without assured additional funding for linkage to care, and making patients bear the costs of increased HIV screening in health care settings.

The objective of this research was to obtain a systematic, balanced, and in-depth evaluation of the CDC recommendations from informed sources. In order to accomplish this objective, we attempted to capture the full breadth and complexities of the participants’ perspectives. Therefore, we conducted semi-structured, qualitative interviews with academicians, members of advocacy groups, clinicians, policymakers, and researchers who had voiced their opinions in the media and professional and lay literature in an attempt to conduct a systematic ethical analysis of the CDC recommendations. First, we asked respondents to identify the potential benefits and the possible risks or harms posed by the revised HIV testing policies. Second, we asked whether respondents viewed each policy recommendation as either fulfilling or violating moral responsibilities to patients. Third, we asked whether each policy recommendation respected or a violated patient rights. By using a qualitative methodology, we ensured that the participants were allowed to describe any nuances of their perspectives without any preconceived suggestions. We provide an accounting of the predominant themes. Our aim was to inform the continuing debate on the implementation of the CDC HIV testing recommendations for the health care setting.

## Methods

### Study design

This manuscript reports on a qualitative analysis of responses to semi-structured interviews with 25 members from the following fields: US HIV advocacy, clinical or social care, policy, or research. Each individual had commented on the 2006 CDC HIV testing recommendations in the media or lay or professional literature. The methods described for this manuscript are similar to those reported in our previous paper [32], except as noted here. The Rhode Island Hospital Institutional Review Board approved this study.

### Study population

In August 2007, we performed a search of MEDLINE, Philosopher’s Index, SocIndex, the internet, and medical and public health journal websites for all articles, commentaries, editorials, press releases, publications, research, and statements about the 2006 CDC HIV testing recommendations. From this search, 164 documents or websites met this criterion. From these, 55 authors or persons quoted were identified, and constituted the group of potential study participants*.* US government officials were excluded from participation because of a potential for conflict of interest.

Based on their remarks, the 55 potential study participants were categorized as either critics (or at least voicing concerns) or supporters of the CDC’s recommendations. These two groups were then subdivided by respondent occupation. This sorting yielded five strata of participants: supportive advocates (n = 11), concerned advocates (n = 11), supportive academicians/clinicians/researchers (n = 15), concerned academicians/clinicians/researchers (n = 9), and local or state government (non-federal) officials (n = 9). Using these strata, a list of potential participants was generated.

In accordance with recommendations for conducting qualitative research [33-35], we chose an *a priori* sample size of 25 participants (five participants for each of the five strata). A research assistant, who was not involved in the interviewing process, contacted potential participants by email, letter, and telephone and invited them to participate in a telephone interview. When extending the invitations to potential participants, the research assistant attempted to achieve a gender-balanced and geographically-diverse participant sample. Invitations were extended to potential participants until five persons in each stratum were interviewed. No incentives were provided to participants.

### Protocol development

The study authors developed a protocol for the semi-structured interviews. The semi-structured interview design was chosen in order to allow participants to answer the stated questions in a comprehensive fashion and without preconceived biases. The protocol contained questions germane to this analysis and included the following HIV testing policy topics: (1) nontargeted HIV screening, (2) making HIV screening similar to screening for other treatable conditions, (3) increasing HIV screening without assured additional funding for linkage to care, and (4) making patients bear the costs of increased HIV screening in health care settings. The protocol contained open-ended questions that asked respondents for their opinions on each the four policy topics according to four ethical domains: (1) the benefits of the change in HIV testing policy; (2) the risks or harms of the change; (3) how the change in testing policy fulfills or violates ethical responsibilities of a health care provider to administer appropriate medical care to patients; and (4) how the change in testing policy respects or violates patient rights. These four ethical domains were chosen by our multidisciplinary group of investigators (from the fields of clinical medicine, public health, epidemiology, philosophy-bioethics, and survey research methodology) and reflect the most common approaches to the ethical evaluation of policies: consequentialist and rights approaches. Of the four familiar principles of bioethics, beneficence and non-maleficence are thought to be grounded in a consequentialist framework, while duties to respect autonomy and duties of justice are often associated with a rights-based approach to morality [36].

### Interview administration

The interview questions were part of a larger study that gathered participants’ perspectives on the 2006 CDC HIV testing recommendations, as described in our previous article. Prior to the telephone interviews, participants received a copy of the 2006 CDC HIV testing recommendations and a brief synopsis of the study and study topics. Two of the study authors [MJW and RCM] conducted the interviews with each person performing approximately half of the interviews. The interviewers were blinded to participant strata and participants’ previous comments in the lay and medical literature. All interviews were conducted via telephone from October 2007 through August 2008.

At the start of each interview, each participant was provided with a description of the nature of the study, the topics under discussion, the questions that would be asked, and definitions of any terms used in the interviews. Interviewers quoted the relevant CDC HIV testing recommendation prior to initiating a discussion about that HIV testing policy topic. Participants were asked to confine their responses to ethical considerations about each HIV testing policy, as opposed to the practical implementation of the policy. Participants were prompted as necessary to focus their answers on the specific policy recommendation under discussion, and reminded to avoid commenting on the remaining HIV testing methods, policies, or the recommendations as a whole. Participants giving short answers to the questions were prompted to elaborate. Interviewers did not otherwise provide any commentary or feedback to the participants. All interviews were audio-recorded and later transcribed and de-identified by a research assistant who was not involved in the interviews.

### Analysis

A qualitative content analysis was performed on the de-identified transcripts without regard to participant strata. A quantitative analysis of the responses was not performed, due to the sample size, methods of administration, purposive stratification of respondents, and goals of the planned analysis. The transcribed responses were reviewed and coded by the two interviewers. The two interviewers identified themes, sub-themes, and sub-sub-themes implicit in the participant responses. Two secondary reviewers [MTC and MAC], who had not conducted the interviewers, independently reviewed separate random samples of 50% of the transcripts for accuracy and thoroughness of the data extraction and selection of themes, sub-themes, and sub-sub-themes. Afterwards, the primary and secondary reviewers discussed their findings; inconsistencies were discussed and reconciled.

## Results and discussion

### Nontargeted HIV screening

#### *Benefits of nontargeted HIV screening*

Respondents stated that making HIV screening nontargeted would identify more new infections. As one respondent summarized this benefit, “it may capture more undiagnosed individuals and offer them a chance to access care at an early stage and (a chance) at prevention services.” Per respondents, this benefit in turn leads to positive downstream effects, including providing earlier access to medical care and preventive services and – through earlier diagnosis – better health outcomes and a reduction in mortality. Second, nontargeted HIV screening broadens the population, setting, and scope of HIV testing, making HIV testing more widely available. This broadening of the population also leads to positive downstream effects, including providing greater education about HIV and HIV testing. Third, nontargeted HIV screening makes more people aware of their status, which, in turn, leads to positive downstream effects such as reducing future infections through changes in risk-taking behavior for both infected and uninfected individuals (Table 1).

**Table 1:** Themes of nontargeted HIV screening

**Nontargeted HIV Screening**	
***Benefits***	***Risks or Harms***
***Theme***: Identifies more new infections	***Theme***: Tests under suboptimal circumstances
***Sub-theme***: Leads to positive downstream effects of identifying new infections through increased testing	***Sub-theme***: Forces patients to be tested when not ready, or at inappropriate times
***Sub-subtheme***: Provides earlier access to medical care	***Sub-theme***: Increases “pychotrauma” and emotional distress of testing
***Sub-subtheme***: Provides earlier access to preventive services	***Sub-theme***: Increases potential for physical and psychological harm of those tested
***Sub-subtheme***: Reduces mortality and better health outcomes because of earlier diagnosis	***Sub-theme***: Disallows individualization of testing
***Theme***: Broadens the population, setting, and scope of testing, making testing more widely available	***Sub-subtheme***: Causes erosion of physician-patient relationship
***Sub-theme***: Leads to positive downstream effects	***Theme***: Results in poor health care and financial resource utilization
***Sub-subtheme***: Provides greater education about HIV and HIV testing	***Sub-theme***: Results in unnecessary testing because of low yield
***Theme***: Makes more people aware of their status	***Sub-subtheme***: Increases risk of false positives, which leads emotional/financial distress and erosion of physician-patient relationship
***Sub-theme***: Leads to positive downstream effects	
***Sub-subtheme***: Reduces future infections through changes in risk-taking behavior for those with positive or negative HIV test results	***Theme***: Leads to potential for cavalier implementation of testing recommendations
	***Sub-theme***: Ignores need for population-specific HIV testing procedures, e.g., age-related, cultural ***Sub-theme***: Further reduces likelihood that clinicians will review patient’s sexual risk
***Theme***: Improves efficiency and performance of testing techniques by clinical providers	
***Theme***: Reduces patient barriers to testing	***Theme***: Results in negative downstream consequences of increased testing
***Theme***: Reduces stigma of HIV testing	***Sub-theme***: Overburdens health care system with newly diagnosed people needing treatment
***Theme***: Improves the structural framework and health care system for HIV testing	
	***Theme***: Creates resentment towards testing by patients and caregivers
	***Sub-theme***: Results in patient avoidance of health care
	***Theme***: Increases financial and emotional burden upon those who might not truly require testing
***How does nontargeted HIV screening fulfill responsibilities to patients?***	***How does nontargeted HIV screening violate responsibilities to patients?***
***Theme***: Serves public health needs	***Theme***: Increases testing without clear provision of meeting needs of those who are infected
***Sub-theme***: Provides public health care needs of community	
	***Theme***: Creates conflicts with parents over testing of adolescents
***Sub-theme***: Positively impacts health of individual though reduced transmission	
	***Theme***: Increases costs and emotional burdens because of unnecessary testing and increased likelihood of false positives
***Theme***: Obtains more information about patient’s health and health needs	
***Theme***: Conducts necessary screening for maintaining and promoting health	
***Theme***: Identifies unrecognized HIV infections or risks for infection	
***Theme***: Broadens screening to previously untested populations	
***Theme***: Destigmatizes HIV testing	
***How does nontargeted HIV screening respect patients’ rights?***	***How does nontargeted HIV screening violate patients’ rights?***
***Theme***: Improves health care outcomes through screening for a treatable condition	***Theme***: Fails to individualize care
	***Sub-theme***: Focuses on public health care
***Theme***: Informs patient about their health	
	***Sub-theme***: Does not provide patient-centered testing experience
***Theme***: Expands testing to those who might not have been tested otherwise	
	***Theme***: Increases testing without clear provision of meeting needs of those who are infected
***Theme***: Meets state-of-art medical care	
***Theme***: Increases likelihood patients will be tested for HIV	
***Theme***: Destigmatizes HIV testing	
***Theme***: Identifies unrecognized HIV infections	

Finally, respondents noted that nontargeted HIV screening improves efficiency and performance of screening (by avoiding potential pitfalls of targeted screening, such as providers incorrect assessments of risk or need for testing); reduces patient barriers to testing; reduces the stigma of HIV testing; and improves the structural framework and health care system for HIV testing.

### Risks or harms of nontargeted HIV screening

Five concerns emerged regarding the risks or harms associated with nontargeted screening. First, respondents were concerned that testing might be conducted under sub-optimal circumstances. Nontargeted HIV screening might force patients to be tested when they are not ready, or at inappropriate times. It might increase the “psychotrauma” and emotional distress of testing itself, increase the potential for physical or emotional harm after testing (e.g. from domestic abuse), disallow individualization of testing, and erode the physician patient relationship.

Second, nontargeted HIV screening may lead to poor health care and financial resource utilization. Respondents stated that unnecessarily screening large numbers of people, particularly those with a low chance of being HIV infected, would result in a low yield per test. In the same light, when screening large numbers of people who may not be at risk, there is an increased risk of false positives, which leads to emotional and financial distress and erosion of the patient-physician relationship.

Third, nontargeted HIV screening has the potential to be implemented in a cavalier fashion. As one respondent commented, “People sort of relax their standards.” Respondents were concerned that nontargeted HIV screening ignores the need for population-specific HIV screening procedures, such as fashioning different processes for adolescents and other demographic groups. In addition, clinicians might not review patient’s sexual risks and behaviors at the time of the health care interactions.

Fourth, there are negative downstream consequences of increased testing as a result of nontargeted screening. In particular, some respondents were concerned that nontargeted screening would overburden the current health care system with newly diagnosed people needing treatment.

Finally, nontargeted HIV screening places an increased financial and emotional burden upon those who might not truly require testing, and as a result, patients and caregivers might resent or try to avoid HIV testing. Similarly, some respondents were fearful that patients might avoid health care all together if they thought they could not seek medical attention without being tested.

### Fulfilling responsibilities to patients and respecting patients’ rights with nontargeted HIV screening

Respondents perceived that nontargeted HIV screening improves the general public health of the community by improving the health of those individuals who are infected. Further, it also improves the health of those individuals who might have potentially been exposed because of the reduced transmission associated with more people knowing their HIV status. Reasons cited for this belief were that: nontargeted HIV screening increases the likelihood that individuals will be tested for HIV; obtains more information for patient’s about their health and health needs; conducts necessary screening for maintaining and promoting health; improves health care outcomes through screening for a treatable condition; identifies unrecognized HIV infections or risks for infection; broadens screening to previously untested populations; meets state-of-the-art medical care; and destigmatizes HIV testing.

### Violating responsibilities to patients and patients’ rights with nontargeted HIV screening

Some respondents were concerned that nontargeted HIV screening could create conflicts with parents over the testing of adolescents. They were also concerned that nontargeted HIV screening increases costs and emotional burdens because of unnecessary testing and the increased likelihood of false positives. They were concerned that nontargeted screening leads to an increase in testing without a clear provision of how to meet the needs of those who are found to be infected. Finally, nontargeted HIV screening was perceived to focus too much on public health, fail to individualize care, and not provide for a patient-centered experience.

Some respondents commented that nontargeted HIV screening could potentially violate responsibilities to patients and patients’ rights if patient needs and readiness were not assessed prior to testing, if proper informed consent was not used, or if poor processes and procedures were implemented. One respondent also commented that the 2006 CDC recommendations for nontargeted HIV screening did not include children younger than 13- or adults older than 64-years-old, and, therefore, did not address the health needs of these populations.

Some respondents also remarked that nontargeted HIV screening focuses more on the health care needs of the society as a whole, instead of the individual. In this light, nontargeted HIV screening neither fulfilled nor violated patients’ rights. In addition, one respondent commented that nontargeted HIV screening involves an avoidance of individual discussions with patients, which underscores the tendency of clinicians to generally avoid frank discussions about sex and sexuality with patients.

### Making HIV screening similar to screening for other treatable medical conditions

Opinions about the recommendation to make HIV screening similar to screening for other treatable conditions were diverse. Some respondents viewed this recommendation as a positive change. They believed the new policy would promote the view of HIV testing as a routine part of maintaining health, eliminate HIV exceptionalism, and eradicate the false assumptions about risk for HIV and associated stereotypes. Others viewed this recommendation negatively. They believed HIV is a distinctly different disease that carries its own stigma. In their opinion, these factors warrant that HIV screening be treated differently from screening for other treatable medical conditions. Other respondents thought that HIV could be treated like other medical conditions, but health care providers should remain respectful of the disease/condition and its implications and uniqueness. Still other respondents were concerned that making HIV screening similar to screening for other treatable medical diseases could lower testing standards, or allow providers to order an HIV test without any safeguards, which might not be optimal care for everyone (Table 2).

**Table 2:** Themes for making HIV screening similar to screening for other treatable medical conditions

**Making HIV screening similar to screening for other treatable medical conditions**	
***Benefits***	***Risks or Harms***
***Theme***: Destigmatizes HIV testing	***Theme***: Ignores exceptional nature of HIV
***Sub-theme***: Destigmatizes who is at risk and removes stereotypes about risk	***Sub-theme***: Leads to persistence of stigma and adverse social, societal problems
***Theme***: Leads to positive downstream affects	
	***Sub-theme***: Underestimates gravity of HIV diagnosis
***Sub-theme***: Identifies more HIV infections	
	***Sub-theme***: Falsely equates HIV with other sexually transmitted diseases
***Sub-theme***: Increases testing utilization	
	***Sub-theme***: Downplays significance of HIV in society
***Sub-theme***: Identifies people earlier in infection	
	***Theme***: Leads to potential for poor or cavalier implementation of HIV testing recommendations
***Sub-theme***: Promotes view of HIV as a treatable, chronic condition	
	***Theme***: Promotes screening by inadequately prepared clinicians
	***Theme***: Encourages unneeded, superfluous testing
***Sub-theme***: Gives an understanding of the true extent of the HIV epidemic	***Theme***: Fails to individualize screening
	***Theme***: Creates conflicts in physician-patient relationships
***Sub-theme***: Improves public health	
***Theme***: Facilitates HIV testing	
***Sub-theme***: Increases provider comfort in offering HIV screening	
***Sub-theme***: Increases patient comfort in being tested for HIV	
***Sub-theme***: Encourages other medical/public health groups to recommend routine HIV screening	
***Sub-theme***: May lead to third-party insurers to pay for routine HIV screening	
***Sub-theme***: Streamlines HIV testing process for providers	
***Sub-theme***: Increases patient comfort in being tested for HIV	
***Sub-theme***: Makes testing more convenient and accessible for patients	
***Theme***: Promotes view of HIV testing as a routine part of maintaining health	
***Theme***: Eliminates HIV exceptionalism	
***Theme***: Eliminates the assumptions about risk for HIV and associated stereotypes	
***Theme***: Normalizes HIV testing in comparison to other screening tests	
***Theme***: Engenders belief that HIV screening is a part of maintaining good health	
***How does making HIV screening similar to screening for other treatable medical conditions fulfill responsibilities to patients?***	***How does *** **making HIV screening similar to screening for other treatable medical conditions *violate responsibilities to patients?***
***Theme***: Conducts necessary screening for maintaining and promoting health	
	***Theme***: Violates obligation to assess patient’s emotional health and safety before testing
***Theme***: Obtains more information about patient’s health and health needs	
	***Theme***: Ignores exceptional nature of HIV
***Theme***: Identifies unrecognized HIV infections and facilitates linkage to care	
***Theme***: Facilitates HIV testing	
***Theme***: Facilitates harm and risk reduction to patients and their contacts	
***How does making HIV screening similar to screening for other treatable medical conditions respect patients’ rights?***	***How does *** **making HIV screening similar to screening for other treatable medical conditions *violate patients’ rights?***
***Theme***: Incorporates a process into good, standard clinical care	***Theme***: Creates a potential for poor or cavalier implementation of HIV testing recommendations
***Theme***: Leads to increased testing, which has positive downstream effects	
***Theme***: Reduces stigma	

### Benefits of making HIV screening similar to screening for other treatable medical conditions

One respondent summarized many of the benefits of making HIV screening similar to screening for other treatable medical conditions as, “I would say that the major benefits are that it streamlines the process, it really demystifies and to me really reduces or removes the stigma of being tested, because it is just part of what we do in providing medical care.” In general, respondents thought that making HIV screening similar to screening for other treatable medical conditions leads to positive downstream affects. These positive downstream effects include: identifying more HIV infections and more people earlier in their infection; increasing testing utilization; promoting a view of HIV as a treatable, chronic condition; providing an understanding of the true extent of the HIV epidemic; and improving public health.

Second, making HIV screening similar to screening for other treatable medical conditions facilitates the HIV testing process and HIV testing in general. Respondents cited that this change in policy: increases provider comfort in offering testing and patient comfort in accepting testing; encourages other medical/public health groups to recommend routine HIV screening; streamlines the HIV testing process for providers; makes testing more convenient and accessible for patients; and leads to third-party insurers to pay for routine HIV screening.

Additionally, making HIV screening similar to screening for other treatable medical conditions promotes the view of HIV testing as a routine part of maintaining health; eliminates HIV exceptionalism; removes the assumptions about risk for HIV and associated stereotypes; normalizes HIV testing in comparison to other screening tests; engenders belief that HIV screening is a part of maintaining good health; destigmatizes HIV testing, reduces stigma about who is at risk for HIV, and changes stereotypes about risk.

### Risks or harms of making HIV screening similar to screening for other treatable medical conditions

The risks or harms of making HIV screening similar to screening for other treatable medical conditions were divided into several themes. First, making HIV screening similar to screening for other treatable medical conditions ignores the exceptional nature of HIV. As one respondent commented, “I think it portrays a colossal misunderstanding of what living with HIV is like … and despite progress on the medical front and in the medical management of HIV, there is still enormous stigma around HIV and people have a really hard time grappling with and assimilating the information and moving forward.” Respondents commented that there is, in fact, a persistence of stigma and adverse social and societal problems with HIV. Making HIV screening similar to screening for other treatable medical conditions underestimates the gravity of being diagnosed with HIV, falsely equates HIV with other sexually transmitted diseases, and downplays the significance of HIV in society.

In addition, making HIV screening similar to screening for other treatable medical conditions: creates a potential for poor or cavalier implementation of HIV testing recommendations; promotes screening by inadequately prepared clinicians; encourages unneeded, superfluous testing; fails to individualize screening; and creates conflicts in physician-patient relationships by potentially having providers routinely order a test that patients do not perceive as needed. One respondent also commented that many patients falsely presume that routine HIV testing has been occurring all along.

### Fulfilling responsibilities to patients and respecting patients’ rights by making HIV screening similar to screening for other treatable medical conditions

Several themes emerged on how making HIV screening similar to screening for other treatable medical conditions might fulfill ethical obligations to patients and respect patents’ rights. Making HIV screening similar to screening for other treatable medical conditions leads to a necessary screening for maintaining and promoting health; incorporates a screening process into good, standard clinical care; facilitates HIV testing; facilitates harm and risk reduction to patients and their contacts; identifies unrecognized HIV infections and facilitates linkage to care of these patients; obtains more information about patient’s health and health needs; leads to increased testing, which has positive downstream effects; and reduces stigma.

### Violating responsibilities to patients and patients’ rights by making HIV screening similar to screening for other treatable medical conditions

Respondents perceived that making HIV screening similar to screening for other treatable medical conditions might violate patients’ rights because it negates an obligation to assess patients’ emotional health and safety before testing; ignores the exceptional nature of HIV; and creates the potential for poor or cavalier implementation of HIV testing recommendations.

### Increasing HIV screening without assured additional funding for linkage to care

There were a few premises and overarching themes that pervaded much of the responses regarding increasing HIV screening without assured additional funding for linkage to care. Some respondents espoused the view, summarized by one respondent, that “if you diagnose HIV-infected individuals, additional money for linkage to care will follow.” These respondents believed that advocacy could only start with testing and diagnosis. They believed that if large numbers of HIV-infected individuals are diagnosed, the health care system would adapt. Another general premise was that some patients – such as those with insurance or access to free HIV care – would benefit from increased testing and diagnosis efforts regardless of whether or not there was additional funding for linkage to care. Respondents stated that there is already screening for medical conditions other than HIV (such as cancer) without assured linkage to care, so HIV screening should not be an exception. Another premise was that the first obligation of clinical providers is to diagnose, and that they should look for treatment options afterwards. A final overarching theme supporting HIV screening without additional funding for linkage to care was that withholding knowledge of a patients HIV status was unethical given that testing technology was available (Table 3).

**Table 3:** Themes for increasing HIV screening without assured additional funding for linkage to care

**Increasing HIV screening without assured additional funding for linkage to care**	
***Benefits***	***Risks or Harms***
***Theme***: Broadens the population, setting, and scope of testing, making testing more widely available	***Theme***: Adds to the burden of an HIV diagnosis
	***Sub-theme***: Further expands social, physical, emotional, psychological, and financial burdens of HIV
***Sub-theme***: Identifies infections earlier	
***Sub-subtheme***: Helps promote better disease management	
	***Sub-theme***: Increases stigma and discrimination
***Sub-subtheme***: Informs patients of their health care status	
	***Sub-theme***: Further expands social, physical, emotional, psychological, and financial burdens of HIV
***Sub-subtheme***: Helps patients advocate for their health care needs	
	***Sub-theme***: Further disenfranchises vulnerable people
***Sub-theme***: Ultimately, allows more patients to receive treatment	
	***Sub-theme***: Increases opportunities for self-harm, self-destructive behaviors health
***Sub-subtheme***: Increases advocacy for improved access to HIV care	
	***Theme***: Emphasizes testing at expense of treatment
***Sub-theme***: Identifies more infections	
***Theme***: Facilitates HIV prevention	
***Sub-theme***: Decreases HIV risk-taking behavior	
***Sub-theme***: Improves patient knowledge of their HIV status	
	***Sub-theme***: Disenfranchises/devalues vulnerable populations
	***Sub-theme***: Discriminates against HIV
	***Sub-theme***: Lowers quality of care to meet demand of testing
	***Theme***: Establishes a lower than ideal and harmful level of care for those with HIV
	***Sub-theme***: Ignores complex needs of HIV care
	***Sub-theme***: Poorer health outcomes because of lack of ability to afford care
	***Theme***: Disincentivizes testing
	***Sub-theme***: Disincentivizes testing by providers
	***Sub-theme***: Disincentivizes testing by patients
	***Theme***: Overwhelms current HIV health care and support resources
***How does increasing HIV screening without assured additional funding for linkage to care fulfill responsibilities to patients?***	***How does increasing HIV screening without assured additional funding for linkage to care violate responsibilities to patients?***
***Theme***: Fulfills obligation to inform patients about their health	
***Theme***: Initiates path to medical care	***Theme***: Incurs harm without significant benefit
***Theme***: Fulfills obligation to diagnose	***Theme***: Is an inappropriate action because testing when treatment exists is not ethical
	***Theme***: Manipulates people into being tested under false pretenses
	***Theme***: Promises hope of benefit without clear intention of treatment
	***Theme***: Reduces quality of access and care as resources are overwhelmed
***How does increasing HIV screening without assured additional funding for linkage to care respect patients’ rights?***	***How does increasing HIV screening without assured additional funding for linkage to care violate patients’ rights?***
***Theme***: Informs patients about their health	***Theme***: Violates right to receive care for a treatable condition
***Theme***: Leads to an expansion of resources for HIV care	***Theme***: Knowingly diagnoses early, but induces harm because patients will suffer without treatment
***Theme***: Empowers patients to take action to improve their health	***Theme***: Violates benefit from information about your health
***Theme***: Increases advocacy for improved access to HIV care	***Theme***: Does not treat medical care and testing on an equal basis
	***Theme***: Violates purpose of testing to lead to medical care and benefits to patient

There were differences in how respondents viewed whether the benefits of screening without assured additional funding for linkage to care outweighed the risks. Respondents who started with the premises that (a) the ethical value of screening is to the individual, and (b) there is an expectation for follow-up care and treatment believed that this policy violates patients’ rights when funding is not assured. On the other hand, some respondents started with the premise that nontargeted screening can be justified by its benefits to society alone. These respondents generally viewed HIV screening without assured additional funding for linkage to care as ethically appropriate because it helped some individuals as well as society as a whole.

### Benefits of increasing HIV screening without assured additional funding for linkage to care

The benefits associated with increasing HIV screening without assured additional funding for linkage to care were grouped into two main themes. First, increasing HIV screening without assured additional funding for linkage to care broadens the population, setting, and scope of testing, thereby making testing more widely available. Under this theme, there were two sub-themes. First, increasing HIV screening without assured additional funding for linkage to care identifies infections earlier, which, in turn, helps promote better disease management, informs patients of their health status, and helps patients advocate for their health care needs. Furthermore, more patients will ultimately receive treatment because identifying more patients creates more opportunities for advocacy efforts to improve access to HIV services. As one respondent said, “I think that you have to go forward anyways with the testing, because the responsibility starts with people knowing their status.” The second benefit theme was that increasing HIV screening without assured additional funding for linkage to care facilitates HIV prevention by (a) improving patient knowledge of their HIV status and (b) decreasing HIV risk-taking behavior.

### Risks or harms of increasing HIV screening without assured additional funding for linkage to care

Some respondents perceived that screening without assured funding for linkage to care adds to the burden of an HIV diagnosis. The decoupling of diagnosis and linkage to care would increase the social, physical, emotional, psychological, and financial burdens of HIV. It would increase stigma and discrimination associated with HIV, further disenfranchise vulnerable people, and increase opportunities for self-harm and self-destructive behaviors. One respondent stated, “You’ve now added to their own personal misery index.”

Second, respondents expressed worry that increasing HIV screening without assured additional funding for linkage to care emphasizes testing at the expense of treatment. This emphasis, in turn, disenfranchises and devalues populations who may benefit from testing but do not have the resources to pay for treatment once they are diagnosed. In addition, increasing testing without assured additional funding for linkage to care potentially floods HIV care clinics and lowers the quality of care of all of those who are HIV-infected to meet the demand of those who are newly diagnosed.

Third, increasing HIV screening without assured additional funding for linkage to care establishes a lower than ideal and potentially harmful level of care for those with HIV. This lower standard ignores the complex needs of HIV care and leads to poorer health outcomes because of a lack of ability to afford care. Finally, testing without assured linkage disincentivizes testing by providers and patients and overwhelms current HIV health care and support resources.

### Fulfilling responsibilities to patients and respecting patients’ rights by increasing HIV screening without assured additional funding for linkage to care

Respondents believed that increasing HIV screening without assured additional funding for linkage to care fulfills responsibilities to patients and respects patients’ rights because it fulfills an obligation by providers to diagnose and inform patients about their health. In addition, it empowers patients to take action to improve their health; initiates a path to medical care; increases advocacy for improved access to HIV care; and, eventually, leads to an expansion of resources for HIV care.

### Violating responsibilities to patients and patients’ rights by increasing HIV screening without assured additional funding for linkage to care

Respondents perceived that increasing HIV screening without assured additional funding for linkage to care violates responsibilities to patients and patients’ rights because it lures people into being tested under false pretenses and does them more harm than benefit. Moreover, it would promise the hope of benefit even though there is no intent to treat. It would violate a patient’s right to receive care for a treatable condition and to have information about his or her health and to benefit from that information. It would defeat the purpose of testing, which is to lead to medical care that benefits the patient. It is unethical to test for a treatable condition and withhold that treatment; it does not treat medical care and testing on an equal basis; and, reduces quality of access and care as resources are overwhelmed by newly diagnosed individuals.

### Making patients bear the costs of increased HIV screening in health care settings

Most respondents believed that it would respect patients’ rights if they were to pay for the HIV test if they had the means to do so; and it would violate patients’ rights to ask them to pay for the test if they did not have the means to do so. Some respondents believed that the cost of HIV testing was cheap when compared to the alternative of not knowing one’s status. However, some respondents acknowledged that this benefit might be more pertinent for those who test positive than those who test negative. Other respondents believed the costs of health care and public health interventions (such as HIV screening) that benefit society should be borne by society (Table 4).

**Table 4:** Themes for patients bearing the costs of increased HIV screening in health care settings

**Making patients bear the costs of increased HIV screening in health care settings**	
***Benefits***	***Risks or Harms***
***Theme:*** Enables pPatients to learn their HIV status and get treatment as needed	***Theme:*** Creates a barrier to testing
	***Sub-theme***: Discriminates against those least able to pay who need testing most
***Theme:*** Encourages patients to “take ownership” of their behavior and health needs	
	***Sub-theme***: Leads to an avoidance of testing because of costs
***Theme:*** Serves as a motivator to decrease risky behavior	
	***Sub-theme***: Encourages self-rationing of testing
***Theme:*** Makes HIV screening similar to screening for other treatable conditions	
	***Theme:*** Leads to question value of HIV testing because of its costs
	***Theme:*** Forces people to pay for something they might not want or need
	***Theme:*** Uses testing as a source of revenue instead of patient benefit
***How does making patients bear the costs of increased HIV screening in health care settings fulfill responsibilities to patients?***	***How does making patients bear the costs of increased HIV screening in health care settings violate responsibilities to patients?***
***Theme***: Identifies patients needing treatment	***Theme:*** Creates a barrier to good health care
	***Sub-theme***: Discriminates against vulnerable populations
***Theme***: Enables public health benefit of testing	
	***Sub-theme***: Decreases demand for testing despite responsibility for having patients tested
***How does making patients bear the costs of increased HIV screening in health care settings respect patients” rights?***	***How does making patients bear the costs of increased HIV screening in health care settings violate patients’ rights?***
***Theme***: Informs people about their HIV status	
	***Theme***: Discriminates by economic and class status
***Theme***: Enables autonomy of choice in medical care	***Theme***: Obviates community-level benefit of screening in society
	***Theme***: Inhibits access to medical services
	***Theme***: Inhibits willingness to undergo screening

### Benefits of making patients bear the costs of increased HIV screening in health care settings

The benefits of making patients bear the costs of HIV testing were that patients would learn their HIV status and get treatment as needed. As one participant stated, “knowledge is power.” This benefit is especially noteworthy for patients who are diagnosed earlier than they otherwise would have. Second, respondents perceived that patients paying for their own HIV test might serve to make them think about their risk for HIV, which could perhaps motivate them to reduce their risk-taking behavior. Finally, respondents noted that making patients bear the costs for their own screening tests in fact makes HIV screening similar to other medical procedures for which patients are responsible.

### Risks or harms of making patients bear the costs of increased HIV screening in health care settings

Respondents highlighted two major risks or harms associated with making patients bear the costs of increased HIV screening. First, this creates a barrier to testing. This barrier can exist because patients may decline testing because they are unable to pay for the test. This consequence was especially worrisome to some respondents because they were concerned that those who cannot afford testing or those who are dissuaded from testing because of its costs could be the same individuals who need testing the most. Accordingly, if patients bear the costs of testing, then they may self-ration testing and test less frequently than needed. As one respondent stated, “It violates (patients’ rights) to the extent that it has an impact on the ability of those who need the services to get them.”

Second, making patients bear the costs of testing might lead to them to question the value of HIV testing because of its costs. Other respondents also stated that making patients bear the costs of HIV testing essentially compells some individuals to pay for something they might not want or need. This possibility was cited as harmful, regardless of whether patients needed to pay for the test themselves or through their insurance. Finally, it would be a waste of money for patients who were at low risk of infection to bear the costs of HIV testing and this would result in poor resource utilization.

### Fulfilling responsibilities to patients and respecting patients’ rights by making patients bear the costs of increased HIV screening in health care settings

Respondents stated that charging patients for the cost of HIV testing is consistent with caregiver responsibilities to patients to test patients and demonstrates respect for patients’ rights to know their HIV status. Since patients can choose or refuse testing, patients are able to exercise autonomy with regard to the medical care they receive.

### Violating responsibilities to patients and patients’ rights by making patients bear the costs of increased HIV screening in health care settings

Respondents stated that making patients bear the costs of HIV testing would violate responsibilities to patients and violate patients’ rights because it would create a barrier to good health care. This violation was especially true for those who need testing and cannot pay for it, such as vulnerable populations. In addition, having patients bear the costs of testing would decrease the demand for testing, which some respondents believed was a violation of patient rights.

Respondents also believed that making patients bear the costs of HIV was potentially discriminative against those without the ability to pay for access to information and care. Further, by discouraging testing, this policy violated society’s rights to be protected against infectious diseases.

## Conclusions

When they first promulgated their recommendations in 2006, the stated goals of CDC were to increase the number of individuals who know their status and to destigmatize HIV and HIV testing. Respondents disagreed on whether or not implementation of the recommendations would have these effects. Nontargeted HIV screening and making HIV screening similar to screening for other treatable medical conditions were often seen by respondents as beneficial when these two recommended policy changes were viewed as accomplishing the CDC goals of increasing the number of patients tested and destigmatizing HIV testing and HIV. On the other hand, nontargeted HIV screening and treating HIV screening similar to screening for similar treatable conditions were seen as detrimental when they were perceived as potentially ignoring the exceptional nature of HIV and HIV testing, and when they lead to “cavalier” testing. In order to reconcile these conflicts, further research or attention might be given to ensuring that when routine, nontargeted HIV screening is implemented, it is done so in a fashion that ensures that patients are not tested without their knowledge or permission. It is likely that these two recommended policy changes would be viewed as ethical if they increased the number of people tested without leading to testing of individuals who were unaware of or did not agree to testing.

Nontargeted HIV screening and making HIV screening similar to screening for other treatable medical conditions were also seen as detrimental if scarce resources were wasted when perhaps more targeted approaches to testing could instead be implemented. These findings highlight the ongoing debate in terms of research utilization between nontargeted and targeted testing strategies. At least one study in an emergency department setting has shown that targeted HIV screening may find just as many new positive cases as nontargeted testing while using fewer resources [37]. To add further sophistication to the debate surrounding targeted vs. nontargeted testing, it may be true that targeted testing may save resources, and nontargeted testing may reduce the stigma of HIV and HIV testing. While the importance of these perhaps competing priorities has not been determined, ongoing and future experience and research will guide policy makers moving forward.

Finally, much of respondents discussion on nontargeted HIV screening and making HIV screening similar to screening for other treatable medical conditions disagreement highlighted the still present debate about HIV exceptionalism, and whether HIV and HIV testing should still be treated differently than other medical diseases. Those respondents who viewed HIV testing as an exceptional disease expressed opinions which generally reflected a greater degree of individualization and protection of patients’ rights when approaching HIV testing, while other respondents who believed that HIV was no longer an exceptional disease, believed the 2006 CDC HIV testing policies were in line with the destigmatization of HIV and HIV testing. Over the last couple of decades, with the advances in treatment and gradual destigmatization of HIV, testing for HIV has become more “routine.” Nevertheless, this topic remains quite contentious and vocal advocates continue to argue both for and against making HIV testing similar to screening for other treatable medical conditions.

When analyzing participants’ responses on the topics of increasing HIV screening without additional funding for linkage to care and making patients bear the costs of increased HIV screenings, we found that participants’ viewpoints often varied depending on their underlying premises about HIV testing. Participants who approached HIV testing from the “knowledge is power” position generally thought that HIV testing was a beneficial intervention and should be performed regardless of assured linkage to care or who would bear the costs. On the other hand, participants who were more concerned about the exceptional nature of HIV and HIV testing were consequently defensive of patients’ individual rights. While they agreed that HIV testing was, in general, good, they also opined that HIV testing was a violation of patient autonomy or rights when patients were tested for HIV without being provided the opportunity for treatment. External funding for costs of testing and/or linkage to care would mitigate the criticisms against patients bearing the costs of testing and increasing HIV testing without additional funding for linkage to care. Finding ways to provide external funding – through private, state, or federal grants, insurance coverage, or other methods – might be a top priority for those invested in scaling up testing [38]. It should be noted, however, that these CDC recommendations for HIV testing in clinical settings were published in September 2006. Since this time, the US Congress has passed the Affordable Care Act and the United States Preventive Services Task Force (USPSTF) has given HIV screening a Grade A recommendation to HIV screening. The Affordable Care Act will likely increase the proportion of individuals living in the US with health insurance. And, the Grade A recommendation from the USPSTF compels insurance companies to reimburse HIV screening. Therefore, compared to 2006, it is likely that fewer patients overall will bear the costs of HIV testing in the coming years. There are still issues yet to be determined, however, such as how frequently HIV screening will be reimbursed, how HIV testing will be reimbursed in the context of bundled patient charges, etc.

### Limitations

This qualitative analysis has several limitations. By their nature, qualitative analyses do not necessarily weigh the strengths of one participant’s argument against another, or try to quantify how many respondents agreed with one perspective or another. Therefore, this manuscript does not attempt to conclude which viewpoints are more valid or should be given more weight.

## Competing interests

The authors have no competing interests.

## Authors’ contributions

MJW participated in the conception and design of the study, the data collection and coding, and writing of the manuscript. RCM participated in the conception and design of the study, the data collection and coding, and writing of the manuscript. MTC provided the moral frameworks informing the survey design and participated in the coding of data, and the writing of the manuscript. MAC participated in the design of the study, the coding of data, and the writing of the manuscript. All authors read and approved the final manuscript.

## Pre-publication history

The pre-publication history for this paper can be accessed here:

http://www.biomedcentral.com/1472-6939/14/46/prepub
